# Mixed methods instrument validation: Evaluation procedures for practitioners developed from the validation of the Swiss Instrument for Evaluating Interprofessional Collaboration

**DOI:** 10.1186/s12913-023-09040-3

**Published:** 2023-01-25

**Authors:** Jean Anthony Grand-Guillaume-Perrenoud, Franziska Geese, Katja Uhlmann, Angela Blasimann, Felicitas L. Wagner, Florian B. Neubauer, Sören Huwendiek, Sabine Hahn, Kai-Uwe Schmitt

**Affiliations:** 1 Bern University of Applied Sciences, School of Health Professions, Bern, Switzerland; 2 Academic-Practice-Partnership of Bern University of Applied Sciences and Insel Gruppe, Bern University Hospital, Bern, Switzerland; 3 University of Bern, Institute for Medical Education, Department for Assessment and Evaluation, Bern, Switzerland

**Keywords:** Validation study, Surveys and questionnaires, Mixed methods, Interprofessional collaboration, Healthcare delivery

## Abstract

**Background:**

Quantitative and qualitative procedures are necessary components of instrument development and assessment. However, validation studies conventionally emphasise quantitative assessments while neglecting qualitative procedures. Applying both methods in a mixed methods design provides additional insights into instrument quality and more rigorous validity evidence. Drawing from an extensive review of the methodological and applied validation literature on mixed methods, we showcase our use of mixed methods for validation which applied the quality criteria of congruence, convergence, and credibility on data collected with an instrument measuring interprofessional collaboration in the context of Swiss healthcare, named the Swiss Instrument for Evaluating Interprofessional Collaboration.

**Methods:**

We employ a convergent parallel mixed methods design to analyse quantitative and qualitative questionnaire data. Data were collected from staff, supervisors, and patients of a university hospital and regional hospitals in the German and Italian speaking regions of Switzerland. We compare quantitative ratings and qualitative comments to evaluate the quality criteria of congruence, convergence, and credibility, which together form part of an instrument’s construct validity evidence.

**Results:**

Questionnaires from 435 staff, 133 supervisors, and 189 patients were collected. Analysis of congruence potentially provides explanations why respondents’ comments are off topic. Convergence between quantitative ratings and qualitative comments can be interpreted as an indication of convergent validity. Credibility provides a summary evaluation of instrument quality. These quality criteria provide evidence that questions were understood as intended, provide construct validity, and also point to potential item quality issues.

**Conclusions:**

Mixed methods provide alternative means of collecting construct validity evidence. Our suggested procedures can be easily applied on empirical data and allow the congruence, convergence, and credibility of questionnaire items to be evaluated. The described procedures provide an efficient means of enhancing the rigor of an instrument and can be used alone or in conjunction with traditional quantitative psychometric approaches.

## Background

Questionnaire development comprises procedures that are qualitative and quantitative. For instance, generating items to represent a construct involves qualitative processes. These include a literature review and conducting expert interviews or focus groups to extract relevant dimensions and develop items that capture them [1, 2]. In a further qualitative process, developed items are judged by experts whether they capture all aspects of a dimension and are well understood by prospective respondents [3]. This is sometimes supplemented by a quantitative assessment of whether the items are relevant and understandable, such as in the example of the Content Validity Index [4–6]. When an initial draft of the instrument has been developed, a qualitative cognitive pre-test is advised [7]. Quantitative procedures then come into play as the battery of items is tested against statistical criteria, such as Cronbach’s alpha coefficient to demonstrate internal consistency [8] or bivariate correlation coefficients to demonstrate construct-related and criterion-related validity [9, 10]. Despite the fact that both qualitative and quantitative procedures are involved in instrument development [11, 12], quantitative methods may be overemphasised [13] and qualitative methods neglected [14].

These circumstances contribute to the perception that instrument development is bound to its methodological tradition, wherein only quantitative approaches are appropriate for developing quantitative instruments [15]. The neglect of qualitative methods holds untapped potential for validation and opens up new means of collecting evidence of construct validity [16]. Given that qualitative and quantitative procedures are part of instrument development and assessment, we propose that their mix can provide additional insights into instrument quality that go beyond the contributions of a mono-method alone [15]. Specifically, we propose that a mixed methods (MM) approach to instrument validation (IV) will enrich the process with more rigorous validity evidence [17].

We develop procedures and illustrate the potential of MM analysis for IV using an instrument measuring interprofessional collaboration (IPC) in the Swiss healthcare context, called the Swiss Instrument for Evaluating Interprofessional Collaboration (SIPEI). IPC in healthcare is understood as the joint efforts of workers from different healthcare professions to provide high quality comprehensive care to patients, families, and communities across settings [18]. The importance of IPC has been recognised by the World Health Organization (WHO) since the 1970s, with research showing that IPC may have a positive impact on patient satisfaction, length of hospital stay, and access to healthcare services [19]. It may also increase the flow of information between professions [20] and workplace satisfaction of health professionals [21, 22].

In the following, we describe our study’s contribution to the instrument validation literature. This is followed by theoretical frameworks for IV and exemplar studies, which we will use to derive validation criteria. Our study demonstrates the utility of MM in IV using sample items of SIPEI to illustrate. We begin by reviewing the literature on MM validation frameworks and validation studies that use quantitative and qualitative methods. We derive criteria and procedures applicable for our IV, given the data collected and the time constraints imposed by our project. Our procedures provide researchers constrained by time, budget, and limited data with a means of enriching an IV through MM.

### Theoretical frameworks for mixed methods validation

Mixing multiple quantitative methods for IV can be traced as far back as Campbell and Fiske’s [23] seminal paper using multitrait-multimethod analysis, which some methodologists view as having formalised the use of multiple methods for validation [15, 24, 25] and even as laying the groundwork for MM research [26]. Multitrait-multimethod analysis, however, does not include any qualitative assessment. With the advent of MM, an overarching approach to instrument development and validation became available that combines quantitative and qualitative methods.

Among the theoretical developments, Dellinger and Leech [16] proposed a unified validation framework (VF) which provides guidance for construct validation by suggesting elements of validity evidence to consider within a MM framework. The authors review existing terminology on validity from the quantitative, qualitative and MM literature and suggest four new quality criteria which can provide information on the validity of a study. Among the criteria, they introduce the concept of a ‘foundational element,’ which refers to researchers’ understanding of a construct or phenomenon. Second, their concept of ‘inferential consistency’ refers to the degree to which a study’s findings agree with previous research. Third, citing Messick [27], they introduce a utility/historical element, which uses past utilization of an instrument as indication of construct validity. Fourth, the authors propose a ‘consequential element’, wherein an instrument’s or study findings’ socially acceptable use is regarded as evidence of ‘consequential validity’.

A second framework, proposed by Onwuegbuzie et al. [15], is a meta-framework that prescribes the use of validation procedures. It consists of a 10-phase process called “Instrument Development and Construct Validation” (IDCV) to optimise quantitative instrument development. Using the different types of validity as starting point (e.g., structural validity, convergent validity, etc.), the authors propose corresponding ‘crossover analyses’, which supplement the traditional analyses associated with various types of validity. Crossover analyses use qualitative methods to analyse quantitative data, and quantitative methods to analyse qualitative data. The framework contains separate quantitative and qualitative analysis phases, but also phases where both methods are combined in crossover analysis. One crossover analysis phase is qualitative-dominant, and another phase is quantitative-dominant. These procedures are designed to enhance instrument fidelity, which encompasses an instrument’s appropriateness or utility.

Another notable framework was proposed by Adcock and Collier [28] and applied in a MM instrument validation [17]. Adcock and Collier [29] discussed the lack of shared standards for quantitative and qualitative research. They proposed a shared framework for establishing validity that uses quantitative and qualitative methods. It distinguishes four levels a researcher progresses through when developing an instrument and defines tasks between levels that lead a researcher to transition between levels. The starting point, Level 1, is the background concept. The task of conceptualization leads to Level 2, the systematised concept, which is derived from a literature review, usually culminating in an explicit definition of the concept being researched. The task of operationalization leads from the systematised concept to Level 3, the indicators. Finally, the task of giving scores to responses leads to Level 4, to scores for each respondent. The framework focuses on a criterion dubbed ‘measurement validity’, which addresses the relationship between the systematised concept and the observations gathered using the instrument. Measurement validity deals with Levels 2–4, i.e., the systematised concept and measured scores. When initial instrument testing has taken place, a revision can be undertaken by working backward through the levels and making refinements. Adcock and Collier [28] distinguished between three types of validation, merging certain types of validation into one category: 1) content validation, 2) content/discriminant validation, and 3) nomological/construct validation and argued that all three forms could be validated using quantitative and qualitative methods.

The presented frameworks are, to our knowledge, the only frameworks to explicitly propose MM for IV [15, 16] or to have been applied in a MM IV [28]. They vary in the degree to which they specify procedures and the degree to which quantitative and qualitative methods are mixed. Dellinger and Leech’s [16] contribution aimed to guide thinking about validity within quantitative, qualitative and MM traditions and compiled a catalogue of quality concepts related to validity within the three traditions. However, it does not suggest specific validation procedures. Onwuegbuzie et al. [15] provided a 10-phase process for instrument development and validation and suggested specific procedures for handling quantitative and qualitative data analysis on their own and in mixed, crossover analyses as part of a 10-phase process. The elaboration of each phase and its application using an actual example of instrument development [29] bridges the gap between the abstractions of the methodological literature and the hands-on procedures of empirical validation literature. Adcock and Collier [28] developed a four-level framework for instrument development that uses quantitative and qualitative methods separately but does not explicitly combine them to enable deeper insights that transcend separate mono-method analyses. Of the frameworks described, Dellinger and Leech’s [16] is the most abstract and least prescriptive, while Onwuegbuzie et al. [15] and Adcock and Collier [28] provide more explicitly practice-oriented frameworks from which specific procedures are more easily derived.

We next present examples of how multiple methods have been applied in validation studies and propose a typology. Our overview demonstrates the application of multiple methods with varying forms of mixing. Some studies apply MM frameworks developed explicitly for validation purposes. Other studies apply separate quantitative and qualitative mono-methods within the same validation study. This literature informed our validation and can provide other instrument developers with practical analytic examples, which can be varied depending on the time, budget, and data available as well as other project constraints [30].

### Overview of studies applying multiple methods

We propose a typology of multiple method validation studies based on how the methods are applied. Exemplar studies for each type are presented. We apply the term “multiple methods” as an overarching term of multi-method studies which encompasses MM. We also classify as “multiple methods” any study that applies multiple quantitative or qualitative strands within the same study or combines the use of quantitative and qualitative methods within the same study, without mixing data, analyses, and results. This inclusiveness ensures that even studies that might not be “sufficiently mixed” or have the philosophical grounding of MM can be considered for their potential contribution to IV. This is useful, as what constitutes MM has been defined in different ways by leaders in the field and has been part of the MM discourse [24]. Some of these leaders have recognised the inconsistencies between various definitions of MM [31] and have expressed support to continue the discussion on MM’s evolving definition [31, 32].

Multiple-method validation studies can be grouped into three categories: 1) studies that explicitly apply one of the MM frameworks specifically for validation, 2) studies that apply a general-purpose MM design within a validation study (e.g. convergent parallel design, explanatory sequential design) [33], 3) studies that apply quantitative and qualitative methods within the same study but do not mix them. We classify an approach as MM when the study contains quantitative and qualitative analyses, integrates the data and findings to enhance breadth and depth of understanding [24], and is guided by a philosophical stance/worldview [34]. Otherwise, we classify a study as multiple methods.

### Studies that apply a mixed methods framework specifically for validation

We did not find a study that used Dellinger and Leech’s [16] Validation Framework (VF) in instrument development or validation. However, we found a literature review based on the VF. Hales [35] applied the VF to criticise studies guided by culturally responsive teaching and critical race theory. Qualitative, quantitative, and MM elements of the studies were reviewed, and VF criteria were applied to evaluate their quality.

An application of Onwuegbuzie et al.’s [15] 10-phase IDCV process can be found in Koskey et al.’s [36] validation of the Transformative Experience Questionnaire. In this study, the quantitative component using Rasch models provided evidence for content-related and construct-related validity. The qualitative component used cognitive interviews to uncover potential issues with the survey format, item wording, and response scale. The validation procedures that are applied and the validity evidence collected are embedded and described within the 10-phase IDCV process.

### Studies that apply a general-purpose mixed methods design within a validation study

Enosh et al.’s [37] development, testing, and validation of the Client Violence Questionnaire applies a sequential MM design. The questionnaire is designed to measure client violence experienced by social workers. The development and validation process has four stages. The first stage comprises semi-structured qualitative interviews to discover forms of client violence, followed by the three stages as suggested by Schwab [38], which correspond to common procedures in quantitative instrument development. They included a stage in which single items are formulated, another stage combining the items into a scale, and a final stage wherein a psychometric assessment is conducted. This resulted in a 14-item self-report instrument measuring the frequency social workers encounter four types of client violence. Enosh et al. [37] argue that the addition of a qualitative component as a distinct stage, together with the more traditional components of quantitative instrument development, contributed to the fidelity, appropriateness, and utility of the instrument [39].

Luyt’s [17] validation of an instrument measuring male attitude norms expanded upon Adcock and Collier’s [28] framework and applied it in a convergent parallel design. His modified framework described a cyclical process of instrument design that alternated between measurement development, validation, and revision, using MM to achieve its objectives. While Adcock and Collier’s [28] framework describes qualitative and quantitative methods that can be used in parallel to collect the same type of validity evidence, e.g. for content validation or convergent validation, they do not explicitly propose a mix of data and findings. Luyt’s [17] validation approach, however, performed an explicit method mix and grounded its procedures within the philosophical foundations that characterise MM [25, 34, 40].

### Studies that apply quantitative and qualitative methods but do not mix them

An objective similar to the study of Enosh et al. [37] was pursued by Waldrip and Fisher [41] in developing and validating the Cultural Learning Environment Questionnaire, wherein a qualitative component was used to enrich quantitative psychometric procedures. The instrument’s purpose was to measure culturally sensitive factors that affect learning environments. After quantitative analyses, a qualitative component provided further evidence of construct validity. Students were asked about their perceptions of the instrument, using qualitative interviews. This included determining how students interpreted scales of constructs and items. The students’ statements were compared whether they corresponded to the authors’ intentions. Although this study combined qualitative and quantitative components, it lacks the statement of a philosophical stance or worldview to indicate from which ontological, epistemological, and axiological perspective the study is to be understood [34]. It also lacks the statement of a specific MM design. More importantly, the qualitative data are not directly compared to any quantitative data. Rather, the qualitative data are compared with other qualitative data.

A further example of multiple methods without mixing is a study by Groenvold et al. [42] which re-examined the validity of a validated quality of life questionnaire (EORTC QLQ-C30) developed for cancer patients in cancer clinical trials. This study explored whether quantitative questionnaire and qualitative interview responses were consistent. The quantitative questionnaire was administered to breast and gynaecological cancer patients one hour prior to the qualitative interview. Raters listened to audio-taped recordings of the interviews and filled in the most appropriate responses into the quantitative questionnaire based on the interview responses. Afterwards, the two groups of questionnaire responses were quantitatively analyzed. It was argued that consistency in responses would provide evidence that the questions were being understood as intended by the instrument developers. In addition, raters also wrote notes of any issues with the respondents’ understanding of the questions, based on the interviews. This provided information why a patient might indicate not experiencing shortness of breath when, in fact, she did. This patient’s rationale was that the shortness of breath was due to being overweight, rather than being due to cancer. Interview comments gave raters insight which questions might cause misunderstanding and discrepant answers, even when they are understood properly. This study showed two types of multiple method use. First, there was a quantification of interview data by transforming interview responses into quantitative questionnaire responses, which were compared quantitatively with self-administered questionnaire responses. Second, qualitative notes were taken by the raters which provided information why response discrepancies might have resulted. As the two data sources resulted in quantitative ratings and there was no qualitative analysis by means of, e.g., content or thematic analysis, we do not regard this study as using a MM approach. In addition, the philosophical stance/worldview is not elaborated. The aforementioned frameworks are summarised in Table 1.

**Table 1:** Theoretical frameworks and applications of multi-method instrument validation

Theoretical Framework/ Framework Type	Characteristics	Applied by	Type of Method Mix	Application and Procedures
Dellinger and Leech [17] / Mixed Methods	•Unified Validation Framework (VF) •Proposes elements of validity evidence to consider within a MM framework to conduct construct validation •Does not prescribe specific procedures	Hales [36]	Mixed Methods-based literature review, using a MM framework for validation	•Application of VF in a literature review •VF used to critique mixed methods studies on culturally responsive teaching
Onwuegbuzie et al. [16]/ Mixed Methods	•Instrument Development and Construct Validation (IDCV) •Proposes procedures called crossover analyses that correspond to specific types of validity •Crossover analysis applies qualitative methods to analyse quantitative data and vice-versa	Koskey et al. [37]	Mixed Methods, using a MM framework for validation	•Validation of the Transformative Experience Questionnaire •Content- and construct-related validity established using quantitative methods •Qualitative component used cognitive interviews to uncover issues with survey format, item wording, and response scale
Adcock and Collier [29] / Multiple Methods (adaptable into a full mixed methods design)	•Four-level measurement validity framework •Proposes shared standards for establishing validity using quantitative and qualitative methods •Specifies procedures to establish quality criteria	Luyt [18] (adapted into a convergent parallel design)	Mixed Methods, using a general-purpose MM design: Convergent parallel design	•Development and validation of the Male Attitude Norms Inventory-II •Applies Adcock and Collier’s four-level framework •Framework is applied in a cyclical process alternating between measurement development, validation, and revision
None specified	n.a.	Enosh et al. [38]	Mixed Methods, using a general-purpose MM design: Sequential design	•Validation of the Client Violence Questionnaire •4-stage process: 1 QL stage and 3 QN stages •Stage 1: Semi-structured qualitative interviews to discover forms of client violence •Stage 2: Formulation of single items •Stage 3: Combining items into a scale •Stage 4: Psychometric assessment
		Groenvold et al. [43]	Multiple Methods, i.e., combined use of QN and QL methods in the same study without full mixing of data or results	•Re-examination of the validity of the Quality of Life Questionnaire EORTC QLQ-30 •Qualitative interview responses were transformed into quantitative responses and compared with the quantitative questionnaire responses
		Waldrip and Fisher [42]	Multiple Methods, i.e., combined use of QN and QL methods in the same study without full mixing of data or results	•Development and validation of the Cultural Learning Environment Questionnaire •Quantitative psychometric procedures •Qualitative interviews asking about respondents’ perceptions of the instrument, including how they interpreted items and scales •Student perceptions were judged whether they corresponded to the instrument authors’ intended meanings and objectives

Note. QN: quantitative, QL: qualitative

### A purposeful selection of validation methods and criteria

Our review of MM frameworks for validation and exemplar studies of multi and MM approaches to validation suggests criteria and methods that might be employed in a validation study. Considering numerous frameworks and approaches, however, also increases the complexity of an IV. As Bamberger et al. [30] noted, evaluation is often tied to constraints which involve budget, time, data, and politics. Practical limitations substantially shape which kinds of data are feasible to collect and which analyses can be conducted. These circumstances coincide with researchers’ desire to make full use of the data available for instrument enhancement. This calls for an approach that is closely oriented toward specific validation objectives and draws only upon criteria and methods necessary to achieve them. Under time and data constraints, Bamberger et al. [30] suggest that a MM approach can help in elaborating the information in data and confirming findings. MM can also help in obtaining different perspectives by combining analyses from a small number of cases.

In our validation of the Swiss Instrument for Evaluating Interprofessional Collaboration (SIPEI) [43] we had short data collection periods and few hospitals and clinics from which data could be collected. These are circumstances we believe to be common for health research studies. With time, data, and the objective of further optimizing the instrument in mind, a feasible approach to strengthening the validation can entail adding an open-ended question to each question/item containing a quantitative rating scale. This provided our validation study supplementary information that could be compared with the quantitative data. If the statements from both data sources converged [44], it would provide additional evidence that the instrument was measuring what it was intended to measure [41, 42]. As in our own study, researchers validating an instrument can also take field notes during data collection, which can be tapped to provide supplementary information.

The procedures we propose share similarities with cognitive interviewing. Both attempt to elicit information on whether the items were understood as intended. Cognitive interviews gather information on how a respondent interpreted an item, how they constructed their answer, which difficulties they had in answering, and any other information that might provide insight into how the respondent came to provide their answer [45]. Two forms of verbal report methods used in cognitive interviewing are think-aloud and verbal probing [46]. In the think-aloud method, the respondent is asked to explain what they are thinking while answering questionnaire items. Think-aloud was part of the initial testing of the newly developed items of SIPEI [47]. In verbal probing, additional questions are asked to gain further insights into the respondent’s thinking [46]. In this paper, we propose procedures with comparable objectives. The main differences are that, in our MM validation procedures, we gain the inferences from analyses of a respondent’s quantitative and qualitative questionnaire answers. This has the advantage of being more scalable to large samples and minimising the additional time required to collect and analyse data. As a complement to cognitive interviewing, our procedures have the benefit of detecting issues with question design that might have been missed in the smaller sample cognitive interviews. The procedures we developed and cognitive interviewing can both be situated within the Messick validity framework [27, 48], which applies generally to instrument validation and is independent of any MM validation frameworks. Viewed from the perspective of Messick’s framework, our procedures should aim to ascertain whether the questions were understood as intended [49] in order to minimise unwanted variability and provide response process evidence [48]. We elaborate on the elements we take from the literature review in the methods section “Validation Criteria and Procedures.”

### The Swiss Instrument for Evaluating Interprofessional Collaboration (SIPEI)

We illustrate the potential of MM analysis for IV using the data collected in our validation of the Swiss Instrument for Evaluating Interprofessional Collaboration (German: “Schweizerisches InterProfessionalitäts-Evaluations-Instrumentarium», SIPEI) [47]. SIPEI is an instrument consisting of three questionnaires, each available in German, French, and Italian. A specific questionnaire was developed to collect data from patients, staff, and supervisors, respectively, to account for different perspectives on IPC. Intended for use within healthcare institutions, it is designed to be setting-agnostic and applicable independent of the specific healthcare unit, department, or institution. Questions are asked in four domains: 1) actual interprofessional collaboration (items denoted by the prefix IPC and PIPC in the patient questionnaire), 2) interprofessional organization (items denoted by the prefix IPO), 3) interprofessional education (items denoted by the prefix IPE), and 4) impact of interprofessional collaboration (items denoted by the prefix IPC_IMP). Details of SIPEI, its theoretical foundation and development, are described elsewhere [50, 51]. For IV, all closed-ended questions have an associated open-ended question to provide comments. The prompt to elicit comments read “Please enter your comment here:”, which was placed to the right of the quantitative response and above a text box, in which the respondent could enter his/her comments. Placing a comment was not mandatory. The employee and supervisor questionnaires each take approximately 20 min to complete. The patient questionnaire can be completed in approximately 10 min [43].

### Applying mixed methods in instrument validation

Several mixed methods designs can be applied in instrument validation. For instance, when quantitative and qualitative data are collected at the same time, (i.e., in parallel), a convergent parallel mixed methods design can be applied. We employed a convergent parallel mixed methods design to validate and optimise SIPEI.

## Methods

We employ a convergent parallel mixed methods design [17, 33] to analyse quantitative and qualitative questionnaire data collected using SIPEI. In this mixed methods design, quantitative and qualitative data are collected at the same time (i.e., in parallel) for the purpose of testing whether the data converge. Data were collected from staff, supervisors, and patients of a university hospital and regional hospitals in the German and Italian speaking regions of Switzerland. The data are used to test procedures which can be applied to open-ended questions in conjunction with quantitative ratings in a mixed analysis. We also test procedures which can be applied to qualitative open-ended questions on their own. The triangulated data allow evidence of construct validity to be collected as indicated by the criteria of congruence, convergence, and credibility. Our research is informed by a post-positivist philosophical stance/worldview [34], which is defined by a belief in an objective reality that is only imperfectly knowable and subject to researchers’ values and judgments.

### Validation criteria and procedures

With the suggestions from the MM literature and the limitations of our study context to guide our decisions, we lay out a purposeful selection of validation criteria and procedures. They contain the following elements that are commonly found in MM research:

- Citing the MM design employed [25, 34]
- Stating the underlying philosophical stance/worldview [25, 34]
- Providing a legitimation/rationale for the use of mixed methods [16, 44]

In addition, we take elements from our review of the theoretical and empirical MM validation literature, wherein instrument development is seen as a process of continuous improvement [15, 16], a cyclical process [17, 28], and where the overarching goal of validation is to establish evidence of construct validity [16, 27, 52]. Specifically, we included criteria that could be tested on our data and would indicate that the questions were being understood as intended, providing evidence of construct validity:

- Congruence [16] between question/item content and responses in open-ended questions
- Convergence between quantitative and qualitative data [44], specifically the agreement between quantitative ratings and qualitative questions, following Waldrip and Fisher [41] and Groenvold et al. [42]
- Credibility [16], based on the type of response in open-ended questions, inferences drawn from comparing quantitative and qualitative responses, and field notes from patient questionnaire data collection

The criteria we propose, their data requirements, associated analyses as well as advantages and disadvantages are summarised in Table 2.

**Table 2:** Quality criteria and associated analyses

Criterion	Data Requirement	Analysis	Result	Advantages / Disadvantages
Congruence	Item phrasing and open comments	Compare whether comments written in an open-ended comment field match with what the question/item is asking	•Congruent (on-topic) •Incongruent (off-topic) •Unclear (cannot be determined) •Not applicable	+Congruence provides *anindicator the question was understood as intended* +Easy to administer +Only marginally increases questionnaire completion time +Respondents with more to say are given the opportunity; all others can simply skip the comments +Allows respondents to clarify quantitative responses for every item +Simple analytic procedure +More scalable to large samples than cognitive interviewing +Can provide indications of quality issues missed in cognitive interviewing -May require adjustments to questionnaire layout -Open comments not always interpretable -Incongruence is not a definitive indicator that the item was misunderstood
Convergence	Response on Likert-type scale and open comments	Check whether quantitative ratings and comments in comment field agree (e.g., high quantitative rating and positive comments)	•Convergent •Divergent •Unclear (cannot be determined) •Neutral (neither convergent nor divergent) •Not applicable	+Convergence provides *an indicator of convergent validity* +Easy to administer +Only marginally increases questionnaire completion time +Respondents with more to say are given the opportunity; all others can simply skip the comments +Allows respondents to clarify quantitative responses for every item +Simple analytic procedure +More scalable to large samples than cognitive interviewing +Can provide indications of quality issues missed in cognitive interviewing -May require adjustments to questionnaire layout -Open comments not always interpretable -Convergence requires articulate and interpretable comments
Credibility	Open comments	1.1.1.1.Classify what the comment is trying to convey, e.g. conveying a clarification, confirmation, disconfirmation of the quantitative rating, expressing comprehension difficulty or inability to judge	•Clarifying statement •Confirming statement •Disconfirming statement •Cannot judge •Comprehension difficulty	+Credibility uses multiple procedures to provide *a summary evaluation of instrument quality* +Provides additional *indicators of whether the question was understood as intended* +Easy to administer +Only marginally increases questionnaire completion time +Respondents with more to say are given the opportunity; all others can simply skip the comments +Allows respondents to clarify quantitative responses for every item +More scalable to large samples than cognitive interviewing +Can provide indications of quality issues missed in cognitive interviewing +Complements analytic procedures for congruence and convergence -More complex analytic procedure -May require adjustments to questionnaire layout -Open comments not always interpretable -Credibility criterion does not have clear cut-off
	Response on Likert-type scale and open comments	2.2.2.2.Compare quantitative response and qualitative comment. Infer whether the respondent is answering the question as intended	Inference of whether the respondent answered the question as intended	
	Field notes	3.3.3.3.Draw inferences from field notes of staff administering paper-and-pencil questionnaires	Inference of whether the respondent answered the question as intended	

### Analytic Procedures

Several MM analyses and one qualitative mono-method analysis were conducted to provide evidence of congruence, convergence, and credibility. All comment fields with content were coded for analysis [53] by one researcher and reviewed by two other researchers. Inconsistencies in coding were reviewed in discussions.

## Results

We begin with a descriptive analysis of the sample, followed by results presented along the criteria selected to establish evidence of construct validity. We selected illustrative items and comments to demonstrate our analyses. Results from staff and supervisor questionnaires are presented together, as their items are comparable. Results from patient questionnaires are presented separately. We conclude with a summary of suggested adaptations to SIPEI.

A total of 1340 staff and supervisors were invited to participate. 435 staff and 133 supervisors participated, corresponding to a response rate of 42.4%. In addition, 189 patients participated in the survey. Table 3 summarises participant characteristics by hospital, profession, and language.

**Table 3:** Participant characteristics

		**Staff** *N* = 435	**Supervisors** *N* = 133	**Patients** *N* = 189
**Hospital**	AA	42	7	38
	BEL	33	6	-
	BER	295	104	110
	LU	26	1	12
	MU	19	7	15
	RI	20	8	14
**Profession**	Dietetics	8	2	-
	Medicine	25	24	-
	Midwifery	38	2	-
	Nursing	298	53	-
	Occupational Therapy	7	4	-
	Physiotherapy	27	9	-
	Psychology	7	-	-
	Social Work	2	5	-
	Speech Therapy	6	-	-
	No occupation given	17	34	-
**Language**	German	386	129	160
	French	15	3	17
	Italian	34	1	12

### Congruence

Evidence for congruence was collected by testing the match between question/item content and the comments written in the associated open-ended response field.

In this analysis (Table 4), we judged whether comments were congruent (on-topic; corresponding to 15 respondents or 39% of the sample), incongruent (off-topic; 7 respondents, 18%), unclear (3 respondents, 8%), or not applicable (13 respondents, 34%). Comments were judged not applicable when they indicated that the respondent could not make a substantive judgment.

**Table 4:** Staff and supervisor item QN-QL comparison

				**Congruence**	**Convergence**	**Credibility**	
**Seniority**	**Question/Item**	**QN response**	**Comment**	**On-/Off-Topic**	**QN-QL Convergence**	**Response Type**	**QN-QL Inference**
Staff	IPC1: Percentage of treatment plans that are jointly developed by **two different** professions	41–50%	C1: can't say exactly because the question is not very clear to me	n.a.	neutral	Unclear	
	IPC2: Percentage of treatment plans that are jointly developed by **more than two different** professions	cannot judge	C2: Although we have many different professional groups that can be included with a patient, there are very few where you develop something TOGETHER, in my perception it is more that each professional group does its part of the work and documents and tries to adapt the care / treatment as well as possible	on-topic	n.a.	Disconfirming statement	
	IPC3: Relevant changes on the treatment plan are made jointly	somewhat disagree	C3: The time is often missed to adapt the treatment plan to the situation in good time (change from curative to palliative)	off-topic	n.a.	Clarifying statement	
	IPC5: The team ensures good coordination of patient treatment	mostly agree	C4: We don't have a special team from different professions. We just work together. So, I'm not sure how to answer	on-topic	neutral	Cannot judge	
	IPC6: The team members know their own and other members’ responsibilities in patient care	somewhat agree	C5: I seldom find understanding on the part of the doctor if I do not find the opinion of the doctor's plan beneficial	off-topic	n.a.	Clarifying statement	
	IPO2: Enough team meetings for joint discussions	fully disagree	C6: There are three interprofessional discussions per patient. The problem is not frequency, but timing and content	off-topic; response does not relate directly to the question	no	Clarifying statement	Evaluation is not based on frequency, despite question asking specifically about the frequency
	IPO3: Suitable rooms for interprofessional meetings available	somewhat agree	C7: Few meetings between doctors and nurses except in situations of complex clinical case discussions	off-topic; response relates to frequency of meetings not availability of rooms	n.a.	Clarifying statement	
	IPO4: Close proximity of individual workplaces eases exchange of information outside of regular meetings	mostly agree	C8: a Computer question / answer can also be used to communicate with doctor / physio	off-topic; response does not relate directly to offices making it easier to communicate	unclear	Clarifying statement	
	IPO6: Electronic patient record system(s) optimally support(s) collaboration	somewhat agree	C9: We can use the field of interdisciplinary questions / answers to clarify questions with the therapist	on-topic	n.a.	Clarifying statement	
		mostly agree	C10: Entries are not always read by everyone involved due to lack of time or knowledge	on-topic	neutral	Clarifying statement	Problems relate not to the systems per se, but to the available time
Supervisor	IPC2: Percentage of treatment plans that are jointly developed by **more than two different** professions	51–60%	C11: Difficult to assess accurately	unclear	neutral	Cannot judge	
	IPC6: The team members know their area of responsibility in patient care	somewhat agree	C12: Discrepancy between "knowing" something and "orienting oneself to it / sticking to it"	on-topic	yes	Clarifying statement	Statement may indicate that the response may not refer to "knowing", but rather to "sticking to it"

The presented SIPEI items are paraphrased

The analysis of the comments provided us with potential explanations why some respondents’ answers were on-topic, off-topic or neither (not applicable), pointing to potential issues with a question. Off-topic remarks and remarks that were neither on- nor off-topic may indicate that a respondent might be answering questions differently than intended by the questionnaire designers. It may also indicate that the question cannot be answered by the respondent or that the question is not relevant to the respondent.

For instance, one comment indicated that the respondent could not answer the question because it was unclear (Table 4, Comment C1). This comment was classified “not applicable” because it was neither on- nor off-topic.

Several off-topic comments seemed to indicate that the question was not being answered as intended and that the quantitative rating might not actually be a response to the question being asked. The reasons why remarks are off topic might not always be apparent.

In one off-topic comment the respondent remarked that he/she saw different issues that should be asked about, instead of the question being asked (C6). Another respondent commented off-topic about seldom finding understanding on the part of the doctor when they disagreed on the treatment (C5), although the item was about interprofessional team members knowing other team members’ responsibilities regarding treatment. A further comment referred to having “few meetings between doctors and nurses” (C7), although the question was about whether there were suitable rooms for interprofessional meetings. One off-topic comment suggested that a computer could be used to communicate with other professionals (C8), even though the question was about whether office spaces made it easy for interprofessional teams to exchange information. Finally, one comment suggested that the time was often missed to adapt the treatment plan “in good time” (C3), although the question dealt with whether relevant decisions were jointly made in interprofessional teams**.**

Despite being on-topic, one comment expressed inability to answer the question on interprofessional collaboration (C4) for lack of an interprofessional team in his/her area of work. One of the on-topic remarks provided a good indication of why the quantitative rating was “cannot judge,” when asked about the percentage of treatment plans jointly developed by more than two professions. This respondent indicated that there were very few treatment plans that were developed together, despite several professional groups working together.

### Convergence

Evidence for convergence was collected by checking for agreement between quantitative ratings and comments (Table 4). When quantitative ratings and their associated comments converge, it provides an indication of convergent validity. In our data, however, the determination of convergence or divergence was only possible in a few cases. The majority of cases were judged “not applicable,” meaning that the criterion of convergence could not be applied. Many of the cases were judged as neutral, i.e., neither convergent nor divergent.

Convergence between quantitative ratings and their associated comments were found in only 5 of 38 questions/items (13%) (Table 4). However, even fewer comments were divergent. Only 2 comments (5%) were divergent (e.g., Staff IPO2). In 2 comments (5%) it was unclear whether they converged with the quantitative responses (e.g., Staff IPO4), because the comment was either off-topic or could not be clearly associated with the question being asked. 10 comments (26%) were judged as neutral (e.g., Staff IPC1, IPC5) because the comments either indicated that the respondent could not adequately answer the question or could not properly understand the question.

The majority of comments (19 comments, 50%) were judged as not applicable (e.g., Staff IPC 3) because no answer was checked in the quantitative rating, or the quantitative response was “cannot judge”. Other comments were judged not applicable because their responses were off topic (e.g., Staff IPC6) and thus could not be related to the quantitative responses, or the comments indicated that respondents did not provide quantitative responses to questions as they were intended (e.g., Staff IPO2).

### Credibility

Credibility was examined in three different tests. We established one type of evidence for credibility by classifying the responses given in the open-ended fields. Response type classifications are “clarifying statements,” “disconfirming statements,” “comprehension difficulty,” and “cannot judge”. In a further examination of credibility, we compared quantitative ratings and open-ended responses to infer whether questions were understood as intended. In a final test of credibility, for patient questionnaires, observations made during data collection and from eyeballing of questionnaires were written down in field notes. The field notes are used to ascertain whether questions were properly understood.

We present credibility evidence as follows, in three analyses: analysis of response types, inference from comparing quantitative (QN) and qualitative (QL) responses, and from the field notes taken during patient data collection.

#### Response type

The analysis of comments by response type attempts to determine what the respondent is trying to convey. The comments can be classified into four types: clarifying statements, disconfirming statements, statements that express difficulty making a judgment (cannot judge), and statements that express lack of clarity of the question (unclear). Clarifying statements may support the credibility of the quantitative rating by giving an indication why a quantitative rating was chosen, whereas disconfirming statements may give reason to doubt the quantitative rating. When respondents are unable to judge a question or express difficulty understanding it, it may indicate the need to re-evaluate the question’s wording or to provide additional information.

Most comments (13 comments, 34%) were clarifying statements to responses given in the quantitative rating. For instance, on the question regarding whether there are suitable rooms for interprofessional meetings (Staff, IPO3), one respondent marked the checkbox that he/she “somewhat agrees” and commented that there were “few meetings between doctors and nurses” (C7). In another example, one respondent noted that entries were “not always read by everyone” (C10), regarding whether “electronic patient record system(s) optimally support(s) collaboration” (Staff, IPO6).

Only 3 comments (8%) provided disconfirming statements, wherein the quantitative rating indicated “cannot judge” but comments expressed that the respondents in fact made a judgment. For instance, on the question for which percentage of patients a treatment plan is jointly developed by staff of more than two different professions (Staff, IPC2), a respondent commented that “there are very few [cases] where you develop something TOGETHER,” demonstrating that the respondent could in fact make a judgement, despite indicating otherwise in the quantitative rating (C2).

11 comments (29%) expressed that the respondent could not judge, for instance, commenting that the question was “difficult to assess accurately” (C11).

11 comments (29%) expressed comprehension difficulty. For instance, on the question for which percentage of patients a treatment plan is jointly developed by employees of more than two different professions (Staff, IPC1), one respondent commented that he/she “can’t say because the question is not very clear (…)” (C1).

#### QN-QL inference

Drawing inferences by comparing quantitative ratings and qualitative comments can support credibility by providing explanations why the respondent answered in the way he/she did. This analysis can inform how to potentially improve wording of an item. It can also provide information to support substantive theorising and even provide indications if the content domain is not adequately captured by the items.

In 3 cases (8%), qualitative comments indicated that there was a discrepancy between the quantitative response and what the question intended to ask (Table 4). For instance, on the question whether there are enough team meetings for joint discussions (Staff, IPO2), one respondent marked that he/she “fully disagrees,” although the respondent’s qualitative comments indicated there are three interprofessional discussions per patient (C6). The respondent went on to comment that “the problem is not the frequency”, but the “timing and content.” This indicates that the quantitative judgment provided was not in terms of frequency, despite the question asking specifically about the frequency.

In another example, regarding whether the electronic patient record system optimally supports collaboration (Staff, IPO6), a respondent marked the checkbox “mostly agree.” However, in his/her comment the same respondent notes that the “entries are not always read by everyone involved due to lack of time or knowledge” (C10). The comment suggests that the systems themselves were adequate but that the limiting factor was having the time and the knowledge to do so. This indicated that the response did not relate perfectly to the question being asked.

Finally, one respondent answering whether team members know their area of responsibility in patient treatment (Supervisor, IPC6) marked the checkbox “somewhat agree.” This respondent went on to comment that there was a “discrepancy between ’knowing something’ and ‘orienting oneself to it / sticking to it’” (C12). This statement appears to be a clarification of why he/she only “somewhat agrees” and may indicate that he/she was answering the question in terms of whether team members “orient themselves to” or “stick to” their responsibilities. The comment provides an indication that the question may not have been answered as the question originally intended.

#### Inferences from patient questionnaires and field notes

We drew qualitative inferences from patient questionnaires and field notes focusing on whether respondents had understood questions as intended, evaluated through the criteria of congruence and credibility. Specifically, we noted whether questions and comments were congruent, i.e., on- or off-topic. We also drew on field notes to assess whether it could be credibly established that questions had been properly understood. We included all 262 patient comments across 7 items for our analysis and below present two items with particularly illustrative comments (Table 5).

**Table 5:** Inferences from patient questionnaires and field notes

	**Congruence**		**Credibility**	
**Question/Item**	**Off-Topic / Unclear Comments**	**On-Topic Comments**	**Field Notes, Observations**	**Field Notes, Inferences**
PIPC1: The team members treated each other with respect *n* = 22	**Patient treatment**, e.g.: • They explain too little to me as a patient, only partially respond to requests. Don't take me seriously • Only 1 person bad experience. Anyone can have a bad day. Otherwise, super brilliant rescue workers. Staff so brilliant and professional **Unclear**, e.g.: • The team and all employees at [name of hospital] are very, very friendly • have great respect for the nursing staff	**Collaboration**, e.g.: • I cannot judge how these people treat each other • the atmosphere among each other seems very collegial. everyone is on a first name basis	Patient said during survey: “I only see how they interact with each other in the room, but not how they interact with each other elsewhere.”	• Possible indication that patients understand the question differently; i.e. how they are treated • Difficult for patients to assess how interprofessional interaction is outside the patient’s room
PIPC6: What was positive about the collaboration between the team members? (optional) *n* = 108 comments	**All well**, e.g.: • all well • everything is good **Patient treatment**, e.g.: • They treat you with respect when you ask questions • That the nursing staff took enough time to talk to the patients. Which was also very pleasant **Unclear**, e.g.: • the humour that could be felt, always friendly • The work was carried out carefully	**Staff collaboration**, e.g.: • That the therapist helped the doctor change the plaster • That they complement each other partly help / teach if they are beginners or in training	• Many one-word responses • Not always possible to understand if they are referring to interprofessional collaboration or patient treatment • Some remarks appear to indicate patient treatment, e.g. “loving care and friendliness”	• Answers kept very brief • Not always clear whether answers relate to collaboration • Difficult to assess whether patients were describing what they liked about the collaboration or what they liked in general

Item PIPC1 asks about whether the team members that looked after the patient treated each other with respect. Field observations indicated that one patient had commented that he/she could only see how the staff interact with each other in the room, but not elsewhere. Field notes further indicated that it was likely difficult for patients to see any interactions outside of the patient’s room. The notes also showed that some patients misunderstood the question as enquiring about how the staff treated them. An off-topic remark such as “they explain too little to me as a patient” is an example for lack of congruence between question and comment. One patient commented that he/she “cannot judge how these people treat each other,” which illustrates what field notes expressed might be difficult for patients.

PIPC6 is an optional open-ended question that asks the patient what was particularly good about the collaboration between the people looking after him/her. Most comments were off topic and were variations of statements that “all is well” or expressed an evaluation of patient treatment by staff. Some comments were unclear as to who or what was being evaluated, for instance a remark about “the humor that could be felt.” The research notes commented that, due to their brevity, comments were sometimes unclear as to who was being referred to.

### Deriving instrument adaptations

We based our suggestions for instrument adaptations on our findings from MM analyses and one qualitative mono-method analysis. Focusing on the criteria of congruence, convergence, and credibility, we explored to what extent adaptations to the existing items are warranted. Three kinds of adaptations to SIPEI were introduced based on the findings: 1) the addition of a definition, 2) emphasizing certain words within a question by underlining them, and 3) reversing the response scale. We present the adaptations proposed for SIPEI by questionnaire and item. A list of suggested adaptations to the items is presented in Table 6.

**Table 6:** Adaptations to SIPEI

Staff Questionnaire			
**Item No**	**Item**	**Issue**	**Recommendation**
IPC1	Percentage of treatment plans that are jointly developed by **two different** professions	For some respondents: • Question unclear • Term “treatment plan” unfamiliar • Difficulty judging whether two different professions jointly develop a treatment plan	• Add a definition of “treatment plan” • “A treatment plan is a list of all procedures, examinations, and treatments that a patient has to undergo.”
IPC2	Percentage of treatment plans that are jointly developed by **more than two different** professions	For some respondents: • Question unclear • Term “treatment plan” unfamiliar • Difficulty judging whether two different professions jointly develop a treatment plan • “Cannot judge” marked as QN answer, but a QL comment is offered that clearly indicates a judgment	• Add a definition of “treatment plan”
IPC11	Drawing attention to errors in appreciative manner	One comment off topic: • No indication question content was understood, instead making reference to “reports” • Otherwise, no indications of problems with question	• Underline “in an appreciative manner” in the item text to underscore this aspect
**Patient Questionnaire**			
**Item No**	**Item**	**Issue**	**Recommendation**
PIPC1	The team members treated each other with respect	• Was not understood by some patients • More likely to be interpreted as the treatment of the patients by professionals	• Underline “each other” to emphasise to whom the question relates
PIPC6	What was positive about the collaboration between the team members? (optional)	• Question answered with short statements • Unclear whether comments are a general evaluation or relate specifically to the collaboration of staff	• Underline “the collaboration” for emphasis • Make question mandatory
PIPC7	How could the collaboration between the team members be improved? (optional)	• No detailed responses, many single word responses provided • Some statements relate to improving patient care instead of collaboration	• Underline keywords “collaboration between the team members” for emphasis
All Questions		• Respondents sometimes answer positively in the closed questions but express criticism in the associated comment field • Indication of possible social desirability bias	• Reverse response scale: Present negative responses first

#### Staff questionnaire

A definition of the term “treatment plan” should be added to items IPC1 and IPC2, as it was indicated that its meaning was unclear. In IPC11 the words “in an appreciative manner” should be underlined for emphasis, as we discovered that this aspect was often not paid attention to.

##### Supervisor questionnaire

No changes are suggested for the supervisor questionnaire.

##### Patient questionnaire

The comments made in Item IPC1 indicate that not all patients understood it as intended. Often, the question was interpreted as meaning how the professionals treat the patients, rather than how the professionals treat each other. Thus, the words “each other” should be underlined to emphasise to whom the question relates. To reduce response set bias, we suggest reversing the response scale such that negative response options are first presented.

## Discussion

Our study results illustrate the utility of MM for validating a quantitative instrument. These methods provide additional sources of construct validity evidence. We draw upon elements from MM frameworks specifically developed for IV as well as empirical validation studies using multiple and MM. We consolidate our methodological review into the three criteria: congruence, convergence, and credibility, with which specific aspects of our data can be evaluated. We add to the instrument validation literature by demonstrating procedures which can be applied to qualitative open-ended questions on their own and in mixed analysis with quantitative ratings. These procedures can serve both as a stand-alone means of collecting evidence of construct validity as well as a complement to traditional psychometric evaluation.

### Translating frameworks and validation studies into practical methods

Applying elements from MM frameworks in a validation study requires that their high level of abstraction is translated into criteria and procedures that can be applied to data.

We were guided by three validation frameworks in particular. Dellinger and Leech’s [16] framework proposes construct validity as overarching framework encompassing all types of validity evidence, in accordance with Messick [27].This suggests multiple paths to construct validation, which can involve approaches using quantitative, qualitative, and mixed methods. Using their VF can guide thinking on validation and provides a set of criteria that can guide validation practice. Onwuegbuzie et al.’s [15] framework proposes specific procedures, which helps to bridge the gap between methodology and validation practice. Adcock and Collier [28] provided an additional multi-method framework that elaborates conceptual levels and tasks involved in instrument development. Within these three validation frameworks, however, guidance is often abstract and lacks the vital link between quality criteria and specific mixed analytic procedures.

Validation studies using multiple methods and MM can often provide more practical guidance, which is easier to implement, bringing the validation practitioner quicker to practical procedures. Validation studies by Groenvold et al. [42] and Waldrip and Fisher [41] illustrated validation examples that relied less on deep methodological grounding and instead focused on practical aspects of validation. One of their validation steps involved showing that respondents understood the questions as they were intended.

The shortcomings in the validation frameworks highlight the lack of practical guidance for practitioners who wish to gain deeper insights into an instrument than can be provided by psychometric analysis alone. Given that research projects typically face various practical constraints [30], a validation study would benefit from deciding early on which data are feasible to collect, which criteria can be evaluated using them, and which procedures need to be applied.

In our study, we were guided by philosophical considerations based on mixed methods validation frameworks as well as mixed methods theory in general, but focused on the procedures for testing congruence, convergence, and credibility.

### Advantages of the proposed criteria and assessment procedure

Our analysis shows that evaluating congruence between a quantitative questionnaire item and what a respondent writes in the associated comment box can serve as an indicator that the question was understood as intended. Conversely, incongruence may be an indication that a respondent may have understood an item differently than intended, for instance when comments are off topic or when it cannot be clearly decided if the comment is on or off topic.

Convergence between a quantitative rating and its associated comment box can serve as an indicator of convergent validity because the qualitative comment confirms what is being stated in the quantitative measure. This bears similarities to Campbell and Fiske’s [23] conceptualization of convergent validity, which is a confirmation of finding between two independent quantitative measures.

Credibility assessed in three different types of analyses provide a summary evaluation of instrument quality. These analyses support the credibility of the quantitative rating because they may provide indications why a given response was chosen. Thus, these analyses can serve as indicators that the question was understood as intended [54, 55]. This is an important consideration, as the misinterpretation of questions can pose a threat to the accuracy of answers [56].

Advantages of the criteria proposed include that they are simple to administer and evaluate using a questionnaire, requiring only a comment box next to the rating scale or below the item. Their implementation only marginally increases questionnaire completion time, as those respondents who wish to write something can do so, while others can simply skip the comments. The procedure allows respondents to comment and clarify responses on each item. The ease of data collection and the simple analytic procedures allow the proposed mixed methods validation to be more easily scaled to large samples than cognitive interviewing. Thus, the proposed criteria and their procedures can complement cognitive interviewing and, through the larger sample size, may provide indications of quality issues that may have been missed in cognitive interviewing. This makes the procedures particularly useful for new instruments being pretested or undergoing their first psychometric validation.

Disadvantages of implementing the criteria may include the need to adjust the questionnaire layout. It also requires that the comments are interpretable. Lack of clarity of open questionnaire comments is a common issue in survey research and needs to be anticipated as a potential data issue. As mixed analysis involves qualitative analysis, criteria may not have any cut-offs. Thus, even when applying the analytic procedures to establish credibility, for instance, the decision whether a questionnaire answer is credible remains a judgment call to be made by the researcher.

We encountered item non-response for comments as a particularly prevalent issue in our study. Andrews [57] found that item non-response may be a greater issue for open-ended questions compared to closed-ended ones. He also found that dissatisfied employees or customers are more likely to respond to open-ended questions and use comment boxes to vent their frustrations. This may explain some off-topic comments we gathered in our study which expressed criticism but did not directly relate to the question being asked. We also found respondents contradicting themselves in their quantitative response and comment. Contradictory statements from the same respondent within the same questionnaire was previously found in hospital patient surveys [58]. It was suggested that it does not imply the question is being misunderstood, rather that patients may have negative comments to make about topics that were not part of the questionnaire, or that patients have negative comments but do not adjust their quantitative ratings. Despite the fact that we administered general comment questions, which are more likely to be answered than explanation-seeking questions [59], the cognitive effort required by our open-ended request for comment may have increased the non-response rate [60]. For instance, it is possible that the cognitive effort to produce a response was high due to the request’s lack of specificity. Another explanation might be that the use of the phrase “Please enter your comment,” rather than asking about whether the respondent had “any thoughts” might have raised the barrier for providing a response because requesting for “a comment” to be entered may be easily interpreted as being asked to write down if “they have something to say” to the researchers. The generic request for comment may have also made it appear less binding to provide one. To address these possible reasons for item non-response in the comment boxes, we propose rephrasing the request for comment as follows: “Do you have any other thoughts on the question you just answered? Please let us know!”

We highlighted the additional data, analysis, and complexity involved in a mixed methods validation, which may help to explain why uptake among instrument developers has been modest so far. We believe it is likely that the lack of easy to follow procedures and the many different, ambiguous quality concepts make a mixed methods validation more daunting to attempt than standard psychometric evaluation. This paper highlights some simple analytic procedures requiring only little additional data, which may help address some of the issues keeping practitioners away from using mixed methods for validation.

### Minor adaptations in preparation for future data collection

Our analyses of qualitative comments, alone and in mixed analysis with quantitative data, suggest that the questions of SIPEI were mostly understood as intended. Accordingly, adaptations to SIPEI were suggested sparingly. Adaptations were focused on making questions clearer by adding definitions [56] and underlining keywords to emphasise key aspects [61, 62]. These changes are unlikely to fundamentally change the instrument’s psychometric properties, but will rather help to reduce unwanted variability [48]. This has provided a refined instrument which can be retested for further psychometric evaluation.

### Limitations

Traditional psychometric analyses were not within the scope of this paper. Thus, the SIPEI instrument’s performance cannot be judged based on the information presented. The mixed methods validation analyses were constrained by missing responses in the qualitative comments. This limited the ability to show convergence. Furthermore, the questionnaires collected were predominantly German language questionnaires. We only collected 15 French and 34 Italian language questionnaires due to the limited hospital access imposed by the COVID-19 pandemic. This reduced the evidence for the French and Italian versions of the questionnaire. Furthermore, samples were obtained from a limited set of participating hospitals. Data collection spanned only two months for the patient survey and three months for the staff survey, limiting the number of questionnaires that could be obtained. More questionnaires could have likely been obtained given a longer data collection period. Our analyses relied on qualitative data from comment boxes and field notes. It is probable that a more expansive data collection strategy, for instance through additional cognitive interviews or focus groups, would have yielded more depth and breadth of data. Finally, no explicit instructions were given on which comments were expected in comment boxes. This likely broadened the variety of comments and reduced the converging validity evidence that might have been collected.

## Conclusion

MM approaches can provide insights into an instrument’s quality and can be used on their own and in conjunction with traditional quantitative psychometric approaches to establish evidence of construct validity. Our approach suggests procedures and criteria that are closer to the empirical data and provides practical examples of how the criteria of congruence, convergence, and credibility can be applied to collect construct validity evidence. This can provide research teams constrained by time, budget, and limited data with an avenue of enriching an IV through MM without necessarily requiring more data.

## Data Availability

Data sets analysed in the current study are available from the corresponding author on reasonable request.

## Abbreviations

AB: Angela Blasimann
FG: Franziska Geese
FN: Florian B. Neubauer
FOPH: Swiss Federal Office of Public Health
FW: Felicitas L. Wagner
IDCV: Instrument Development and Construct Validation
IPC: Interprofessional Collaboration
IPO: Interprofessional Organisation
IV: Instrument Validation
JAG: Jean Anthony Grand-Guillaume-Perrenoud
KU: Katja Uhlmann
KUS: Kai-Uwe Schmitt
MM: Mixed Methods
SHa: Sabine Hahn
SHu: Sören Huwendiek
SIPEI: Swiss Instrument for Evaluating Interprofessional Collaboration
VF: Validation Framework
WHO: World Health Organization

## References

[1] Artino AR, La Rochelle JS, Dezee KJ, Gehlbach H (2014). Developing questionnaires for educational research: AMEE Guide No. 87. Med Teach.

[2] Ricci L, Lanfranchi JB, Lemetayer F, Rotonda C, Guillemin F, Coste J (2019). Qualitative Methods Used to Generate Questionnaire Items: A Systematic Review. Qual Health Res.

[3] Beck K (2020). Ensuring content validity of psychological and educational tests the role of experts. Frontline Learn Res..

[4] Polit DF, Beck CT (2006). The content validity index: are you sure you know what's being reported?. Critique and recommendations Res Nurs Health.

[5] Polit DF, Beck CT. Nursing research : generating and assessing evidence for nursing practice. Tenth edition. ed. Philadelphia: Wolters Kluwer Health; 2017. p. 784.

[6] Glarcher M. Kritische Würdigung des Content Validity Index. Eine Methode zur Quantifizierung der Inhaltsvalidität. Pflegewissenschaft. 2018(9/10):422–9.

[7] Prüfer P, Rexroth M (2000). Zwei – Phasen – Pretesting.

[8] Cronbach LJ (1951). Coefficient Alpha and the Internal Structure of Tests. Psychometrika.

[9] Swank JM, Mullen PR (2017). Evaluating Evidence for Conceptually Related Constructs Using Bivariate Correlations. Meas Eval Couns Dev.

[10] Westen D, Rosenthal R (2003). Quantifying construct validity: Two simple measures. J Pers Soc Psychol.

[11] Zumbo BD. Validity as Contextualized and Pragmatic Explanation, and Its Implications for Validation Practice. Concept of Validity: Revisions, New Directions and Applications. 2009:65–82. https://psycnet.apa.org/record/2009-23060-004.

[12] Chinni ML, Hubley AM. A Research Synthesis of Validation Practices Used to Evaluate the Satisfaction with Life Scale (SWLS). In: Zumbo BD, Chan EKH, editors. Validity and Validation in Social, Behavioral, and Health Sciences. Social Indicators Series. Heidelberg: Springer; 2014. p. 35–66. https://link.springer.com/chapter/10.1007/978-3-319-07794-9_4.

[13] Swanepoel I, Kruger C (2011). Revisiting validity in cross-cultural psychometric test development: A systems-informed shift towards qualitative research designs. Sajp-S Afr J Psychi.

[14] Chan EKH, Munro DW, Huang HS, Zumbo BD, Vojdanijahromi R, Ark N. Validation Practices in Counseling: Major Journals, Mattering Instruments, and the Kuder Occupational Interest Survey (KOIS). In: Zumbo BD, Chan EKH, editors. Validity and Validation in Social, Behavioral, and Health Sciences. Social Indicators Series. Heidelberg: Springer; 2014. p. 67–87. https://link.springer.com/chapter/10.1007/978-3-319-07794-9_5.

[15] Onwuegbuzie AJ, Bustamante RM, Nelson JA (2010). Mixed Research as a Tool for Developing Quantitative Instruments. J Mixed Methods Res.

[16] Dellinger AB, Leech NL (2007). Toward a Unified Validation Framework in Mixed Methods Research. J Mixed Methods Res.

[17] Luyt R (2012). A Framework for Mixing Methods in Quantitative Measurement Development, Validation, and Revision: A Case Study. J Mixed Methods Res.

[18] WHO WHO. Framework for Action on Interprofessional Education & Collaborative Practice. Geneva; 2010. http://apps.who.int/iris/bitstream/handle/10665/70185/WHO_HRH_HPN_10.3_eng.pdf?sequence=1. 21174039

[19] Reeves S, Pelone F, Harrison R, Goldman J, Zwarenstein M (2017). Interprofessional collaboration to improve professional practice and healthcare outcomes. Cochrane Database Syst Rev..

[20] Martin JS, Ummenhofer W, Manser T, Spirig R (2010). Interprofessional collaboration among nurses and physicians: making a difference in patient outcome. Swiss Med Wkly.

[21] Kalisch BJ, Lee H, Rochman M (2010). Nursing staff teamwork and job satisfaction. J Nurs Manag.

[22] Korner M, Wirtz MA, Bengel J, Goritz AS (2015). Relationship of organizational culture, teamwork and job satisfaction in interprofessional teams. BMC Health Serv Res.

[23] Campbell DT, Fiske DW (1959). Convergent and discriminant validation by the multitrait-multimethod matrix. Psychol Bull.

[24] Johnson RB, Onwuegbuzie AJ, Turner LA (2007). Toward a Definition of Mixed Methods Research. J Mixed Methods Res.

[25] Creswell JW, Plano Clark VL (2018). Designing and Conducting Mixed Methods Research.

[26] DeCuir-Gunby JT, Osborne JW (2008). Mixed Methods Research in the Social Sciences. Best Practices in Quantitative Methods.

[27] Messick S (1995). Validity of psychological assessment: Validation of inferences from persons' responses and performances as scientific inquiry into score meaning. Am Psychol.

[28] Adcock R, Collier D (2001). Measurement Validity: A Shared Standard for Qualitative and Quantitative Research. The American Political Science Review.

[29] Bustamante RM, Nelson JA, Onwuegbuzie AJ (2009). Assessing Schoolwide Cultural Competence: Implications for School Leadership Preparation. Educ Adm Q.

[30] Bamberger M, Rugh J, Mabry L (2012). RealWorld Evaluation.

[31] Tashakkori A, Creswell JW. The new era of mixed methods. Sage Publications; 2007. p. 3–7. https://journals.sagepub.com/doi/pdf/10.1177/2345678906293042.

[32] Creswell JW, Tashakkori A, editors. How do research manuscripts contribute to the literature on mixed methods? London, England: Sage Publications Sage UK; 2008. p. 115–20. https://journals.sagepub.com/doi/pdf/10.1177/1558689808315361.

[33] Creswell JW, Plano Clark VL (2011). Designing and Conducting Mixed Methods Research.

[34] Tashakkori A, Teddlie C (2010). SAGE Handbook of Mixed Methods in Social & Behavioral Research.

[35] Hales PD (2016). Changing perspectives: Validation framework review of examples of mixed methods research into culturally relevant teaching. Multicultural Education Review.

[36] Koskey KLK, Sondergeld TA, Stewart VC, Pugh KJ (2018). Applying the Mixed Methods Instrument Development and Construct Validation Process: The Transformative Experience Questionnaire. J Mixed Methods Res.

[37] Enosh G, Tzafrir SS, Stolovy T (2015). The Development of Client Violence Questionnaire (CVQ). J Mixed Methods Res.

[38] Schwab DP, Staw BM, Cummings LL (1980). Construct Validity in Organizational Behavior. Research in Organizational Behavior.

[39] Leech NL, Onwuegbuzie AJ (2010). Guidelines for Conducting and Reporting Mixed Research in the Field of Counseling and Beyond. J Couns Dev.

[40] Brannen J, Brannen J (1992). Combining qualitative and quantitative approaches: An overview. Mixing Methods: Qualitative and Quantitative Research.

[41] Waldrip BG, Fisher DL (2000). The Development and Validation of a Learning Environment Questionnaire Using Both Quantitative and Qualitative Methods. The Journal of Classroom Interaction.

[42] Groenvold M, Klee MC, Sprangers MA, Aaronson NK (1997). Validation of the EORTC QLQ-C30 quality of life questionnaire through combined qualitative and quantitative assessment of patient-observer agreement. J Clin Epidemiol.

[43] Schmitt K-U, Geese F, Grand-Guillaume-Perrenoud JA, Linhart M, Hahn S, Uhlmann K, et al. Förderprogramm «Interprofessionalität» des Bundesamts für Gesundheit. M17: Anwendung und Optimierung des Schweizer Interprofessionalitäts-Evaluations-Instrumentariums SIPEI. Bern: Swiss Federal Office of Public Health; 2020. https://www.bag.admin.ch/dam/bag/de/dokumente/berufe-gesundheitswesen/Interprofessionalitaet/Forschungsberichte1/studie-m17-bfh-anwendung-sipei.pdf.download.pdf/Studie%20M17_Evaluation%20SIPEI_BFH_Schlussbericht.pdf.

[44] Greene JC, Caracelli VJ, Graham WF (1989). Toward a Conceptual Framework for Mixed-Method Evaluation Designs. Educ Eval Policy Anal.

[45] Beatty PC, Willis GB. Research synthesis: The practice of cognitive interviewing. Public Opin Q. 2007;71(2):287–311.

[46] Willis GB. Cognitive interviewing revisited: A useful technique, in theory. Methods for testing and evaluating survey questionnaires. 2004:23–43. https://onlinelibrary.wiley.com/doi/10.1002/0471654728.ch2.

[47] Wagner F, Neubauer F, Huwendiek S (2019). Schweizerisches Interprofessionalitäts-Evaluations-Instrumentarium (SIPEI).

[48] Calhoun AW, Scerbo MW. Preparing and Presenting Validation Studies: A Guide for the Perplexed. Simulation in Healthcare. 2022:10.1097. https://journals.lww.com/simulationinhealthcare/Citation/2022/12000/Preparing_and_Presenting_Validation_Studies__A.3.aspx. 10.1097/SIH.0000000000000667 35470343

[49] Shankar S, St‐Onge C, Young ME. When I say… response process validity evidence. Medical Education. 2022. https://onlinelibrary.wiley.com/doi/full/10.1111/medu.14853. 10.1111/medu.14853 35688144

[50] Wagner FL, Neubauer FB, Grand-Guillaume-Perrenoud JA, Geese F, Schmitt K-U, Huwendiek S. Development and validity evidence of the Swiss Interprofessional Evaluation Instrument (SIPEI). In preparation.

[51] Neubauer FB, Wagner FL, Lörwald A, Guttormsen S, Huwendiek S. Establishing relevant indicators for the evaluation of the quality of interprofessional collaboration and sharing “lessons learned”. In preparation.

[52] Messick S (1998). Test Validity: A Matter of Consequence. Soc Indic Res.

[53] Elo S, Kyngas H (2008). The qualitative content analysis process. J Adv Nurs.

[54] Collins D (2003). Pretesting survey instruments: An overview of cognitive methods. Qual Life Res.

[55] Rickards G, Magee C, Artino AR (2012). You Can't Fix by Analysis What You've Spoiled by Design: Developing Survey Instruments and Collecting Validity Evidence. J Grad Med Educ.

[56] Peytchev A, Conrad FG, Couper MP, Tourangeau R (2010). Increasing Respondents' Use of Definitions in Web Surveys. J Off Stat.

[57] Andrews M (2005). editor Who is being heard? Response bias in open-ended responses in a large government employee survey.

[58] Gallan AS, Girju M, Girju R (2017). Perfect ratings with negative comments: Learning from contradictory patient survey responses. Patient Experience Journal.

[59] Zhou R, Wang X, Zhang L, Guo H (2017). Who tends to answer open-ended questions in an e-service survey? The contribution of closed-ended answers. Behaviour & Information Technology.

[60] Fan W, Yan Z (2010). Factors affecting response rates of the web survey: A systematic review. Comput Hum Behav.

[61] Dillman DA, Smyth JD, Christian LM (2014). Internet, phone, mail, and mixed-mode surveys: The tailored design method.

[62] Olson K, Smyth JD. The effect of emphasis in telephone survey questions on survey measurement quality. Int J Soc Res Methodol. 2020:1–21. 10.1080/13645579.2020.1824628. https://www.tandfonline.com/doi/full/10.1080/13645579.2020.1824628.

[63] Swiss Federal Office of Public Health F. Support programme «Interprofessionality in healthcare 2017–2020» 2021 [Available from: https://www.bag.admin.ch/bag/en/home/strategie-und-politik/nationale-gesundheitspolitik/foerderprogramme-der-fachkraefteinitiative-plus/foerderprogramme-interprofessionalitaet.html.

